# The effect of error aversion climate on impoverished leadership

**DOI:** 10.3389/fpsyg.2025.1503447

**Published:** 2025-04-15

**Authors:** Chang-E Liu, Yunfan Liu, Yulei Li, Chenhong Hu, Shan Wang

**Affiliations:** ^1^School of Business Administration, Hunan University of Technology and Business, Changsha, Hunan, China; ^2^College of Business Administration, Hunan University of Finance and Economics, Changsha, Hunan, China; ^3^Business School Nanjing University, Nanjing, Jiangsu, China

**Keywords:** impoverished leadership, error aversion climate, moral disengagement, ego depletion, regulatory focus

## Abstract

**Introduction:**

Impoverished leadership, as a form of unethical leadership behavior, can have a wide range of negative impacts. It not only affects team morale, work efficiency, cohesion, and trust but also directly influences organizational performance, reputation, and the leader’s own career development. However, previous research has rarely explored the antecedents of impoverished leadership.

**Methods:**

Based on social cognitive theory and conservation of resources theory, this study investigated the impact of error aversion climate on impoverished leadership with mixed methodologies (i.e., a scenario experiment and a questionnaire survey).

**Results:**

The results showed that error aversion climate positively influences impoverished leadership; moral disengagement and ego depletion serve as mediators between error aversion climate and impoverished leadership. Besides, regulatory focus moderate the relationship between error aversion climate and moral disengagement (ego depletion). Specifically, when leaders have high prevention regulatory focus and high promotion regulatory focus, the relationship between error aversion climate and moral disengagement (ego depletion) is stronger. Regulatory focus also moderate the indirect effect of error aversion climate on impoverished leadership through moral disengagement (ego depletion). The indirect effect of error aversion climate on impoverished leadership is stronger when leaders have high prevention regulatory focus and high promotion regulatory focus.

**Discussion:**

The findings provide theoretical guidance for interventions to reduce impoverished leadership and offer new insights for promoting organizational sustainability.

## 1 Introduction

Impoverished leadership refers to the behavior where leaders fail to fulfill or inadequately perform their job responsibilities, which is a form of unethical leadership behavior (Li et al., 2024). Two typical examples of impoverished leadership are evading responsibility and ignoring problems (Li et al., 2024). Impoverished leadership can hinder subordinates’ task completion, leading to decreased work efficiency and negatively affecting the collaborative atmosphere among colleagues and the strategic deployment by higher-level leaders (Pless et al., 2022; Griffin et al., 2022). However, the majority of current research focuses on high-intensity unethical leadership behaviors, such as destructive leadership and abusive supervision (Asim et al., 2024; Almeida et al., 2022; Liu et al., 2025). In contrast, low-intensity unethical leadership behaviors, such as impoverished leadership, are often more easily overlooked or denied due to their subtlety and less noticeable nature, receiving far less attention compared to high-intensity behaviors (Almeida et al., 2022). Given the potential negative impacts and the urgency of studying impoverished leadership, it has become a significant topic of interest for both academia and industry.

Existing studies on impoverished leadership have primarily focused on its conceptualization as a form of passive unethical behavior. For instance, Bass and Avolio (1996) positioned it within the managerial grid as a leadership style marked by minimal engagement with both tasks and people. Recent empirical work has expanded this view, linking impoverished leadership to systemic issues such as bureaucratic inertia in public sectors (Shen, 2019) and proceduralism in corporate settings (Fan, 2021). However, three critical gaps persist. First, prior research predominantly examines impoverished leadership through descriptive case studies (e.g., government inaction) rather than testing its antecedents via theoretical frameworks. Second, while high-intensity unethical behaviors like abusive supervision have been extensively explored (Almeida et al., 2022), low-intensity forms such as impoverished leadership remain under-theorized, particularly in organizational contexts. Third, existing explanations rely heavily on individual traits (Alamri, 2023) or structural factors (Belardinelli et al., 2023). Indeed, research by Ahmed et al. (2021) has highlighted that specific dimensions of organizational climates, especially climates emphasizing error aversion, can also act as catalysts for impoverished leadership (Ahmed et al., 2021). However, few scholars have responded to their call for research.

Error aversion climate refers to a shared perception among organizational members regarding the policies, procedures, and practices related to error management, which includes two dimensions: error strain and covering up errors (Van Dyck et al., 2005). In the VUCA (Volatility, Uncertainty, Complexity, Ambiguity) era, businesses face ever-evolving commercial environments, intense competition, and rapid changes that compel them to continually strive for and adapt to high standards of work environments. In this environment, the cost of trial and error for organizational decision-makers increases significantly. They tend to adopt more cautious and conservative strategies to avoid potential risks. The accumulation of this psychological and behavioral tendency gradually leads to the formation and solidification of error aversion climate (Taskan et al., 2022). Therefore, choosing the error aversion climate as a research perspective for the causes of impoverished leadership can better reveal issues in contemporary organizational management contexts. Theoretically, error aversion climate can cause psychological harm to organizational leaders, triggering a series of negative work attitudes or workplace behaviors, like impoverished leadership. These include conservatism due to fear of making mistakes, which leads to reduced psychological safety and continuous anxiety about errors (Yu et al., 2024; Eshet et al., 2024). Undoubtedly, error aversion climate becomes a source of stress. In such an environment, leaders tend to adopt a passive, conservative attitude to maintain the status quo and ensure their own safety and job stability in a complex and volatile environment, thereby leading to impoverished leadership.

On the one hand, social cognitive theory emphasizes the dynamic interplay among cognitive processes, environmental pressures, and behavioral outcomes (Detert et al., 2008). In error aversion climate, leaders face heightened accountability risks, which erode psychological safety and reinforce the belief that “inaction minimizes exposure to errors.” This cognitive narrowing activates moral disengagement—a process by which leaders decouple their behaviors from ethical standards through mechanisms such as externalizing blame and minimizing consequences (Azhar et al., 2024). Through the mediating role of moral disengagement, this study illustrates how environmental pressure drives leaders to rationalize negative avoidance behavior.

On the other hand, conservation of resources theory elucidates the resource-driven pathway to impoverished leadership. Error aversion climate force leaders into chronic hyper-vigilance, depleting cognitive and emotional resources, leading to ego depletion. When resources fall below a critical threshold, leaders adopt low-energy strategies (e.g., task avoidance) to conserve remaining reserves, manifesting as impoverished leadership (Hobfoll et al., 2018). This pathway highlights resource scarcity as a boundary condition: in contexts lacking psychological safety or institutional support, leaders prioritize survival over responsibility. This pathway is theoretically complementary to social cognitive theory: social cognitive theory emphasizes the cognitive distortion of moral judgment, while conservation of resources theory reveals how environmental stress weakens leaders’ ability to fulfill their responsibilities through resource depletion, both of which jointly explain the dual destructive mechanism of error aversion climate on leadership effectiveness.

In addition, individuals are influenced by individual traits (e.g., regulatory focus) in the process of moral disengagement and ego depletion (Baldner and Pierro, 2024; Asfar et al., 2019). Regulatory focus reveals that due to differences in motivational regulatory tendencies, individuals adopt different self-regulatory strategies to avoid potential risks or pursue positive outcomes (Baas et al., 2011). Specifically, regulatory focus, as an intrinsic trait, can be divided into prevention regulatory focus and promotion regulatory focus. These, respectively, represent tendencies toward loss avoidance and growth pursuit, playing a crucial role in shaping individual behavior patterns (Liu et al., 2020). In addition, compared with stable personality traits, regulatory focus has situational plasticity, which provides a practical entry point for the subsequent design of organizational interventions. Therefore, this study introduces regulatory focus as a key variable, aiming to deeply explore and reveal its boundary-regulating role in an error aversion climate.

In summary, our research may contribute to the existing literature on error aversion climate and impoverished leadership in several ways. First, it redefines the spectrum of unethical leadership by positioning impoverished leadership as a “passive-conservative” counterpart to overtly destructive behaviors. it expands the scope of unethical leadership research by shifting focus from overtly destructive behaviors to passive avoidance patterns, addressing the under explored domain of conservative unethical leadership. Second, the dual-path framework bridges organizational climate (error aversion climate) with psychological processes (moral cognition and ego depletion), addressing calls for multilevel analyses in leadership research (Zhang et al., 2022). Specifically, we extend social cognitive theory by showing how environmental pressures (error aversion climate) distort moral reasoning toward passive harm, and enrich conservation of resources theory by revealing “defensive inaction” as a novel behavioral outcome of chronic resource loss. Third, the moderating role of regulatory focus challenges the simplistic “promotion-positive/prevention-negative” dichotomy, demonstrating synergistic effects where both amplify impoverished leadership.

## 2 Theory and hypotheses

### 2.1 Impoverished leadership

The concept of “Impoverished Leadership” can be traced back to the (1, 1) position in the management grid theory, which represents a leadership style that is neither interested in people nor tasks, exemplifying a typical form of complete inaction (Blake and Mouton, 1964). Bass and Avolio (1996) described impoverished leadership as a behavioral deficit in leadership, where an individual occupies a position but fails to meet subordinates’ expectations, thereby neglecting corresponding responsibilities to some extent. This perspective has been widely accepted among scholars, who further elaborate that impoverished leadership encompasses various types of non-leadership behaviors, such as evading responsibility, failing to respond to issues, being absent when needed, refusing to make decisions, and delaying responses.

While impoverished leadership and laissez-faire leadership share superficial similarities in passivity, their theoretical foundations and behavioral manifestations differ significantly. Laissez-faire leadership, as defined by Bass and Avolio (1996), is characterized by a deliberate avoidance of decision-making and responsibility delegation, often rooted in leaders’ preference for autonomy or trust in subordinates’ capabilities (Bass and Avolio, 1996). In contrast, impoverished leadership emerges from cognitive and resource-driven constraints in high-pressure environments. Unlike laissez-faire leaders who may intentionally refrain from intervention, impoverished leadership exhibit passive withdrawal due to fear of errors and resource scarcity. Empirical studies show that laissez-faire leadership is more related to leader traits (such as low achievement motivation), while impoverished leadership is more driven by organizational climate (Zhang et al., 2022; Chen Y. et al., 2021).

Research on “Impoverished Leadership” in the Chinese context focuses on public officials’ “inaction,” with limited studies defining impoverished leadership within corporate organizations. Shen (2019) notes that inaction among officials stems from their inability to adapt to contemporary professional demands, resulting in passive and arbitrary behaviors. Fan (2021) characterizes this leadership as a tendency for leaders to overthink major issues while neglecting minor ones, leading to superficial engagement in trivial tasks. Building on previous research, this study defines impoverished leadership as a failure to adequately fulfill or perform job responsibilities, resulting in low efficiency, poor quality of work, and incomplete tasks.

### 2.2 Error aversion climate and impoverished leadership

Error aversion climate can affect impoverished leadership in three ways. First, an error aversion climate is inherently a conservative and short-term oriented organizational culture (Klamar et al., 2022). It reflects an organization’s tendency to advocate for maintaining the status quo to avoid risks and to reject bold experimentation (Oliveira et al., 2022). In such an environment, leaders are penalized for even minor mistakes, leading to increased psychological pressure and eroded enthusiasm for their work (Deng et al., 2024). This often results in leaders not fully or adequately performing their duties. Second, leaders are more likely to prioritize safety and avoid taking risks (Farnese et al., 2020). Consequently, this fosters a mindset of “the more you do, the more mistakes you make” and “slacking off to avoid trouble,” making leaders more inclined to engage in impoverished leadership. Furthermore, In an error aversion climate, there exists a negative interaction between leaders and the organization, such as leaders concealing their mistakes to avoid organizational penalties (Plant and Devine, 2003). This negative interaction increases leaders’ anxiety and avoidance behaviors (Chen Y. et al., 2021), which in turn leads to more instances of impoverished leadership. Specifically, the formation of an organizational error aversion climate is often associated with leaders at higher levels of authority (Horvath et al., 2023). Given that individual leaders have limited power to change their work environment, they are more likely to choose tolerance or withdrawal from work (Deng et al., 2024), thereby exhibiting more signs of work withdrawal and impoverished leadership. Based on these discussions and empirical evidence, we propose:

*Hypothesis 1(H1)*: Error aversion climate is positively related to impoverished leadership.

### 2.3 Mediating effect of moral disengagement

Organizational climate is a significant factor influencing moral disengagement (Moore, 2008). So we hypothesizes that error aversion climate can lead to moral disengagement among leaders. On the one hand, as a crucial component of the overall organizational atmosphere, an error aversion climate instills a fear of mistakes among both leaders and regular employees. This fear results in individuals hiding their errors to protect themselves, and even when others’ mistakes are noticed, they are not corrected. Consequently, leaders working in such a climate often experience a sense of helplessness, reduced psychological safety, and increased job insecurity, which can contribute to moral disengagement (Newman et al., 2020). On the other hand, when leaders make mistakes in an error aversion climate, they experience significant pressure and frustration. This increases their work stress and depletes their resources, leading to cognitive strain and, ultimately, moral disengagement (He and Harris, 2014).

Social cognitive theory points that leaders use a set of moral standards to monitor and evaluate their own behaviors (Tenbrunsel and Messick, 2004). If they act contrary to these internal standards, they experience guilt and a sense of conscience (Detert et al., 2008). Generally, individuals act in accordance with their internal moral standards. However, these standards can be moderated or suppressed by self-regulatory mechanisms. Moral disengagement is a process through which individuals suppress their internal moral standards via self-regulation, and it can manifest in three forms (Bandura, 2002). Leaders often use these forms to rationalize and justify their negative behaviors. Specifically, in cognitive restructuring, leaders may interpret impoverished leadership as a form of delegation aimed at developing subordinates, fostering talent within the organization, and thereby portraying their impoverished leadership in a positive light (Li et al., 2025). Furthermore, in neglecting or distorting outcomes, error aversion climate shapes leaders’ interpretations and evaluations of the organization. Leaders may perceive such a climate as indicating that senior management discourages mistakes, leading them to believe that impoverished leadership is a means of avoiding errors and aligning with authoritative expectations. Lastly, in terms of blame attribution, leaders may attribute the harm caused by their inaction to deficiencies in subordinates’ abilities to adapt promptly, rather than reflecting on their own need to adjust for improved performance (Frese and Keith, 2015).

In summary, social cognitive theory posits that individuals actively reconcile conflicts between their actions and moral standards through cognitive rationalization. In error aversion climate, leaders systematically justify impoverished leadership by making moral disengagement through external attribution of responsibility and minimization of consequences. This mechanism reveals the intentionality behind leaders’ paradoxical behavior of “knowing the right course of action yet refusing to act,” reflecting a deliberate cognitive distortion process. Based on these discussions and empirical evidence, we propose:

*Hypothesis 2 (H2)*: Moral disengagement mediates the effect of error aversion climate on impoverished leadership.

### 2.4 Mediating effect of ego depletion

Error aversion climate, as a negative organizational climate, is one of the important stressors for individuals, which can lead to ego depletion for leaders (Deng et al., 2024). On the one hand, error aversion climate implies low organizational tolerance for errors, resulting in heightened emotional responses such as tension, embarrassment, and frustration among both leaders and ordinary employees when facing errors (Aan Mourik et al., 2023). This consumes individuals’ resources and energy, leading to ego depletion (Muraven and Baumeister, 2000). On the other hand, when leaders make mistakes, they experience fear, anger, and worry, with these negative perceptions becoming more intense in an error aversion climate (Dimitrova et al., 2017). They not only have to address the immediate work tasks but also have to deal with the potential negative consequences and the damaged self-image resulting from the errors (Van Dyck et al., 2005). This means that after making a mistake, leaders face additional demands, further exacerbating their feelings of stress and leading to ego depletion (Gillet et al., 2017).

Ego depletion can explain how individuals cope with a stressful work environment (Baumeister et al., 2018). Notably, individuals in an ego depletion state are more inclined to commit unethical behaviors, such as impoverished leadership (Ming et al., 2020). When leaders experience ego depletion, they show decreased attention and are more prone to shallow cognitive processing, meaning they rely more on intuitive heuristic systems in decision-making (Zhao et al., 2025). As a result, they may tend to make “snap decisions,” leading to a disconnect between decisions and reality, thus failing to fulfill the important organizational decision-making role, which constitutes impoverished leadership. Emotionally, leaders in a state of ego depletion exhibit more negative emotions, such as emotional exhaustion (Yang et al., 2023), making it difficult for them to fulfill their role responsibilities required by the job and tending to engage in impoverished leadership. At the same time, they hold a more pessimistic outlook on the future, tend to have low future orientation, and lack the courage to face challenges (Faralla et al., 2017). Consequently, they are more likely to choose impoverished leadership to avoid potential trouble (Deng et al., 2024).

Conservation of resources theory focuses on the passive resource-depletion logic rooted in the dynamic equilibrium of resource management. Error aversion climate forces leaders into a state of chronic hyper-vigilance, which continuously which continuously consumes cognitive and emotional resources and leads to ego depletion. When resource reserves fall below a critical threshold, leaders prioritize conserving remaining resources by adopting low-energy strategies, such as impoverished leadership. This pathway explains the “willing but unable” paradox, reflecting a passive adaptation mechanism shaped by environmental constraints. Based on these discussions and empirical evidence, we propose:

*Hypothesis 3 (H3): Ego depletion mediates the effect of error aversion climate on impoverished leadership.*

### 2.5 Moderating effect of regulatory focus

Social cognitive theory posits that moral disengagement is a flexible cognitive orientation arising from the dynamic interplay between individuals, their environment, and their behaviors (Eissa and Lester, 2021). We propose that individual traits (e.g., regulatory focus) and environmental factors (e.g., error aversion climate) jointly impact moral disengagement (Moore et al., 2012). Regulatory focus can be categorized into two types, each activating mechanisms for self-interest protection (Baas et al., 2011). Prevention regulatory focus, closely associated with the need for security, results in more cautious behavior, a preference for maintaining the status quo, and a tendency to avoid negative attention (Mühlberger et al., 2022). Leaders with prevention regulatory focus aim to minimize negative outcomes and reduce losses. Consequently, in an error aversion climate, leaders with a strong prevention regulatory focus are more likely to engage in moral disengagement as a self-protective strategy to avoid punishment, negative attention, and to preserve their image.

Promotion regulatory focus is closely related to the need for growth and development, encouraging individuals to take more risks in pursuit of positive outcomes and the maximization of benefits (Valle et al., 2019). However, such pursuits only occur when lower-level needs (e.g., physiological and safety needs) are met, leading to further self-actualization. In an error aversion climate, the safety needs of leaders may not be adequately fulfilled. Consequently, leaders with a high promotion regulatory focus may fall into a “conservatism” mindset, focusing on maintaining the status quo rather than striving for higher goals or personal and organizational growth. This situation can create a gap between the ideal self and the actual self, leading to negative emotions such as frustration (Liu et al., 2020), resulting in resource depletion, particularly of cognitive control resources, which in turn may lead to increased moral disengagement (Lee et al., 2016). Moreover, in an error aversion climate, leaders with a high promotion regulatory focus may set “zero errors” as their ultimate goal. This is mainly because, in such a climate, mistakes are severely punished, and only by avoiding mistakes can they gain the organization’s approval and rewards, achieving personal development. Therefore, highly promotion regulatory focus leaders are more likely to take every possible measure to meet the organization’ s expectations, leading to a higher likelihood of moral disengagement regarding their own behaviors. Based on these discussions and empirical evidence, we propose:

*Hypothesis 4a (H4a)*: Leader’s prevention regulatory focus moderates the positive effect of error aversion climate on moral disengagement, such that the positive effect is stronger for leaders with high prevention regulatory focus than those with low prevention regulatory focus.

*Hypothesis 4b (H4b)*: Leader’s promotion regulatory focus moderates the positive effect of error aversion climate on moral disengagement, such that the positive effect is stronger for leaders with high promotion regulatory focus than those with low promotion regulatory focus.

According to the conservation of resources theory, individuals are generally more sensitive to the loss of resources than to their gain (Hobfoll, 1989). leaders with high prevention regulatory focus tend to maintain safety and stability by setting goals and standards centered around defense. They focus on fulfilling obligations and continually adjust their behavior to meet rules and responsibilities (Valle et al., 2019). In an error aversion climate, these high prevention regulatory focus leaders set error aversion climate as their primary work goal. However, mistakes are inevitable and common in the workplace (Putz et al., 2013). Therefore, when high prevention regulatory focus leaders make mistakes, they experience negative emotions for not meeting their standards (Liu et al., 2020). Additionally, the zero-tolerance environment for errors instills a strong sense of fear, leading to further ego depletion as they try to hide their mistakes (Dimitrova et al., 2017).

For leaders with high promotion regulatory focus, the costs of trial and error increase in an error aversion climate. These leaders, who have a higher risk tolerance, are willing to take risks and try new things, which in turn raises their likelihood of making mistakes. However, mistakes result in severe punishments, depleting more resources and leading to greater ego depletion (Kark and Van Dijk, 2007).

Although leaders with high prevention regulatory focus and high promotion regulatory focus pursue their goals differently, in an error aversion climate, they both interpret errors as threats due to the organization’s low tolerance for errors and their potential mistakes. They perceive the threat of resource loss. Based on these discussions and empirical evidence, we propose:

*Hypothesis 5a (H5a)*: Leader’s prevention regulatory focus moderates the positive effect of error aversion climate on ego depletion, such that the positive effect is stronger for leaders with high prevention regulatory focus than those with low prevention regulatory focus.

*Hypothesis 5b (H5b)*: Leader’s promotion regulatory focus moderates the positive effect of error aversion climate on ego depletion, such that the positive effect is stronger for leaders with high promotion regulatory focus than those with low promotion regulatory focus.

Considering Hypotheses 4a and 4b together, We further predict that regulatory focus will reinforce the relationship between error aversion climate and impoverished leadership.

*Hypothesis 6a (H6a)*: Leader’s prevention regulatory focus moderates the relationship between error aversion climate and impoverished leadership mediated by moral disengagement, such that the relationship becomes more vital in the presence of high prevention regulatory focus.

*Hypothesis 6b (H6b)*: Leader’s promotion regulatory focus moderates the relationship between error aversion climate and impoverished leadership mediated by moral disengagement, such that the relationship becomes more vital in the presence of high promotion regulatory focus.

Considering Hypotheses 5a and 5b together, We further predict that regulatory focus will reinforce the relationship between error aversion climate and impoverished leadership.

*Hypothesis 7a (H7a)*: Leader’s prevention regulatory focus moderates the relationship between error aversion climate and impoverished leadership mediated by ego depletion, such that the relationship becomes more vital in the presence of high prevention regulatory focus.

**Figure 1:**
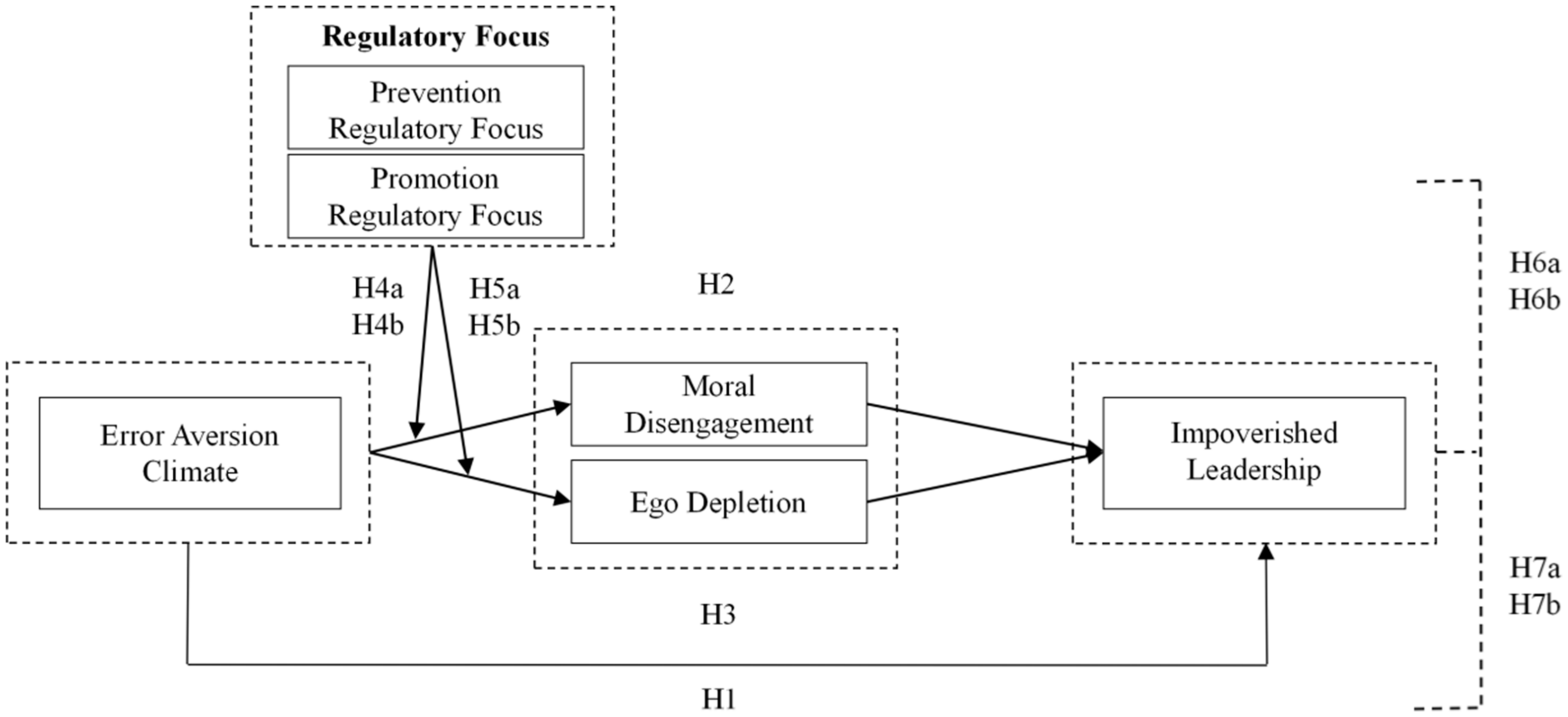
The diagram of theoretical mode.

*Hypothesis 7b (H7b)*: Leader’s promotion regulatory focus moderates the relationship between error aversion climate and impoverished leadership mediated by ego depletion, such that the relationship becomes more vital in the presence of high promotion regulatory focus (See, Figure 1).

## 3 Overview of studies

We conducted two studies (a scenario experiment and a questionnaire survey) to test our theoretical model. Study 1 manipulated the error aversion climate to infer its causal impact on impoverished leadership. To enhance external validity, Study 2 employed both online and offline surveys to examine the full theoretical model. Thus, the mixed design of these two studies provides complementary evidence for our model, addressing both internal and external validity issues.

### 3.1 Study 1: a scenario experiment

#### 3.1.1 Sample and procedures

Study 1 utilizes an industry-acknowledged approach, selecting university students as subjects for the study (Qin et al., 2020). The scenario experiment was conducted through on-site responses, with situational experiments carried out in classes of Human Resource Management, Business Administration, and Marketing. Initially, 160 students were invited to participate in these experiments. After excluding samples that did not pass the attention check, 145 valid samples were obtained. Among the participants, there were 40 men and 105 women, with an average age of 20.34 years (SD = 0.930).

First, before the experiment began, the participants were informed of the rules of the experiment, emphasizing that participation was voluntary and that the data would be used solely for academic research. Second, the participants were randomly assigned to either the “high error aversion climate” group (*N* = 79) or the “low error aversion climate” group (*N* = 66). They were instructed to carefully read the corresponding experimental materials and imagine themselves as the managers of Company G, as described in the materials, for no less than 2 min. Finally, after reading the materials, the participants completed measurements of error aversion climate, moral disengagement, ego depletion, and impoverished leadership, as well as demographic variables such as gender, age, and major. Upon completing the experiment, the participants received a reward as compensation.

#### 3.1.2 Experimental materials

This study developed experimental materials on error aversion climate based on the method developed by Huang et al. (2019). The final materials are as follows:

High error aversion climate group:

“I am a manager at G Company. One common phrase from our ‘head’ is ‘I never make mistakes and do not allow mistakes.’ In our company, inadvertent errors by managers or employees often lead to severe negative consequences. For instance, failing to dispose of garbage in the bin can result in dismissal; a long-time employee was fired for once walking in the motor vehicle lane; a young supervisor was scolded to tears for causing disruptions during a live broadcast due to carelessness; during the early pandemic, an employee was dismissed for attending a gathering, and their department head was also removed from their position..”

Low error aversion climate Group:

“I am a manager at G Company. In our company, if an employee makes a mistake, they do not face severe negative consequences. For example, we once launched a product that resulted in significant losses due to an error. The product manager, fearing dismissal, explained the situation to the ‘head.’ Unexpectedly, the ‘head’ said, ‘You caused the company a loss of 25 million today; I hope you perform better tomorrow.’ Additionally, some young technicians made adjustments to improve the quality of a new product, which unexpectedly resulted in a significant drop in quality. The ‘head’ did not blame them but encouraged them to keep striving to develop better products.”

#### 3.1.3 Measures

We followed the approach of Brislin to do the translation and back-translation process (Brislin, 1970). Unless otherwise noted, all the measures used a five-point Likert-type format, ranging from 1 = “Strongly disagree” to 5 = “Strongly agree.”

##### 3.1.3.1 Error aversion climate

We used the scale developed by Van Dyck et al. (2005), removing items with factor loadings below 0.4, resulting in nine-items. A sample item is “At G Company, I have to make a great effort to focus on certain tasks (Cronbach’*α* = 0.912).

##### 3.1.3.2 Moral disengagement

We used the eight-item scale developed by Moore et al. (2012). A sample item is “At G Company, if people do something because an authority figure told them to, they should not be held responsible” (Cronbach’α = 0.832).

##### 3.1.3.3 Ego depletion

We used the five-item scale developed by Twenge et al. (2004). A sample item is “At G Company, I feel exhausted” (Cronbach’α = 0.895).

##### 3.1.3.4 Impoverished leadership

We used the five-item scale developed by Bass and Avolio (1996). A sample item is “At G Company, managers around me often avoid making decisions and delay responding to urgent issues”(Cronbach’α = 0.838).

#### 3.1.4 Results

##### 3.1.4.1 Manipulation check

This study used an independent samples *t*-test. The results showed that there were 79 participants in the high error aversion climate group and 66 participants in the low error avoidance climate group. The mean score for the high error aversion climate group (*M*_high_ = 3.60, SD_high_ = 0.723) was significantly higher than that of the low error aversion climate group (*M*_low_ = 2.03, SD_low_ = 0.545), with a significant difference (*F* = 5.528, *p* < 0.001). This indicates that the manipulation of the error aversion climate was successful.

##### 3.1.4.2 Main effect hypothesis testing

An independent *t*-test was conducted to test the main effect hypothesis. The results showed that impoverished leadership in the high error aversion climate (*M*_high_ = 3.377, SD_high_ = 0.699) was significantly higher than in the low error aversion climate (*M*_low_ = 2.276, SD_low_ = 0.858), with a significant difference (*F* = 3.146, *p* < 0.001). Therefore, error aversion climate has a significant positive effect on impoverished leadership. Thus, Hypothesis 1 was supported.

##### 3.1.4.3 Mediating effect hypothesis testing

This study employed hierarchical regression analysis to test the hypothesis regarding the mediating effect. The results indicate error aversion climate is significantly positively related to impoverished leadership (*β* = 0.528, *p* < 0.001), moral disengagement (*β* = 0.221, *p* < 0.01) and ego depletion (*β* = 0.724, *p* < 0.001). Moral disengagement is significantly positively related to impoverished leadership (*β* = 0.304, *p* < 0.001). When both error aversion climate and moral disengagement are included in the equation, the effect of error aversion climate on impoverished leadership significantly decreases but remains significant (*β* = 0.485, *p* < 0.001). Ego depletion is significantly positively related to impoverished leadership (*β* = 0.603, *p* < 0.001). When both error aversion climate and ego depletion are included in the equation, the correlation coefficient between error aversion climate and impoverished leadership becomes non-significant (*β* = 0.187, *p* > 0.05). Therefore, impoverished leadership had positive and significant indirect effects on error aversion climate via moral disengagement and ego depletion. Furthermore, the results of the mediation effect test using Bootstrap with 5,000 resamples indicate that the indirect effect of error aversion climate on impoverished leadership via moral disengagement is 0.035, with 95% CI [0.005, 0.075], which does not include 0. The indirect effect of error aversion climate on impoverished leadership via ego depletion is 0.279, with 95%CI [0.158, 0.410], which also does not include 0. Thus, H2 and H3 were supported.

#### 3.1.5 Study 1: discussion

Study 1 used a scenario experiment to examine the causal relationship between error aversion climate and impoverished leadership, and it preliminarily tested the mediating effects of moral disengagement and ego depletion between error aversion climate and impoverished leadership, thereby enhancing the internal validity of the research. To further improve the external validity of the study and to test the moderating effect of regulatory focus, Study 2 utilized a survey method to test the overall model.

### 3.2 Study 2: a questionnaire survey

#### 3.2.1 Sample and procedures

The focus of this study is on impoverished leadership, so the questionnaire was primarily distributed to leaders within various organizations. Data were collected using a combination of online and offline methods. Specifically, on the one hand, the study utilized Questionnaire Star, a data collection platform accepted by the Chinese academic community, to create and distribute the questionnaire via links and QR codes on social media, covering regions including Hunan, Shanghai, and Hubei in China. On the other hand, the study distributed paper questionnaires to MBA students at colleges. To obtain more authentic data, a filter question was set, rendering questionnaires marked as “ordinary employee” under the demographic variable of position as invalid. Additionally, paper questionnaires were screened out if they contained many unanswered questions. A total of 425 paper and electronic questionnaires were distributed, resulting in 400 valid responses from leaders. Therefore, the effective response rate was 94.12%.

The study demographics are as follows. Among participants, 51.5% were male, and 48.5% were female. 52.5% were aged between 26–30 and 31–35. The education level of participants included bachelor’s degree (65.5%), master’s degree (18.8%), PhD (2.2%) and other (13.5%). Regarding their positions, grassroots managers (58.5%), mid-level managers (29.5%), and senior managers (12%) (Table 1).

**Table 1:** The information of samples demographic variable (*N* = 400).

Demographic		Frequency	Percentage
Gender	Male	206	51.5
	Female	194	48.5
Age	Under 25	71	17.8
	26–30	115	28.7
	31–40	146	36.5
	41–50	54	13.5
	Over 51	14	3.5
Education	Bachelor’ s Degree	262	65.5
	Master’s Degree	75	18.8
	PhD	9	2.2
	Other	54	13.5
Position	Grassroots managers	234	58.5
	Mid-level managers	118	29.5
	Senior managers	48	12
Tenure	Less than 1 year	54	13.5
	1–5 years	175	43.75
	6–10 years	84	21
	11–15 years	51	12.75
	More than 15 years	36	9.0
Enterprise category	State-owned enterprise	94	23.5
	Private enterprise	172	43
	Foreign-funded enterprise	16	4.0
	Other	118	29.5

#### 3.2.2 Measures

The same scales from Study 1 were used to measure error aversion climate (Crobanch’s *α* = 0.876), moral disengagement (Crobanch’s α = 0.908), ego depletion (Crobanch’s α = 0.916), and impoverished leadership (Crobanch’s α = 0.881). To obtain more authentic responses, Study 2 utilized a projective method for measuring impoverished leadership, asking participants to evaluate the impoverished leadership around them as an indirect reflection of their own inaction. The instructions included, “The following describes some work behaviors of leaders around you. Please choose based on your understanding of the actual situation.”

##### 3.2.2.1 Regulatory focus

We used the scale developed by Lockwood et al. (2002), which includes two dimensions and a total of seven items. The “promotion regulatory focus” dimension has four items, a sample item is “I often think about how to achieve good results” (Cronbach’α = 0.791). And the “prevention regulatory focus” dimension has three items, a sample item is “I always worry about not meeting my work goals” (Cronbach’α = 0.811).

##### 3.2.2.2 Control variables

Chen X. et al. (2021) suggest that variables such as the leader’s gender, tenure, and the nature of the organization can significantly impact impoverished leadership. Additionally, position level is also an important factor influencing leadership behavior. Therefore, this study includes six variables—gender, age, and position, among others—as control variables to minimize their impact on the data results and research conclusions.

#### 3.2.3 Results

##### 3.2.3.1 Descriptive statistics

Table 2 presents the means, standard deviations, and correlations of all variables. The results indicate that error aversion climate is significantly positively related to moral disengagement (*β* = 0.591, *p* < 0.01), ego depletion (*β* = 0.488, *p* < 0.01), and impoverished leadership (*β* = 0.413, *p* < 0.01). Additionally, moral disengagement is significantly positively related to impoverished leadership (*β* = 0.551, *p* < 0.01), and ego depletion is significantly positively related to impoverished leadership (*β* = 0.453, *p* < 0.01). These results provide preliminary data support for the subsequent hypotheses.

**Table 2:** Descriptive statistics and correlation coefficients of variables.

Variables	Mean	SD	1	2	3	4	5	6	7	8	9	10	11
1.Gender	1.49	0.50											
2.Age	2.96	1.60	−0.133**										
3.Education	2.10	0.64	0.048	−0.046									
4.Position	2.54	0.70	−0.063	0.318**	0.046								
5.Tenure	2.60	1.14	−0.137**	0.689**	−0.019	0.293**							
6.Enterprise category	2.80	1.77	0.024	0.018	0.111*	−0.109*	0.004						
7.Impoverished leadership	2.24	0.89	0.031	−0.174**	0.060	−0.075	−0.122*	−0.061					
8.Error aversion climate	2.78	0.74	−0.057	−0.098	0.039	−0.102*	−0.078	0.045	0.413**				
9.Moral disengagement	2.03	0.78	−0.148**	−0.121*	0.040	0.005	−0.051	−0.102*	0.551**	0.591**			
10.Promotion regulatory focus	3.57	0.72	−0.011	0.065	−0.014	0.103*	0.033	0.053	0.006	0.127*	0.031		
11.Prevention regulatory focus	2.79	0.92	−0.043	−0.068	−0.085	−0.032	−0.117*	−0.002	0.336**	0.461**	0.413**	0.206**	
12.Ego depletion	2.63	0.92	−0.020	−0.07	0.001	−0.036	−0.055	−0.026	0.453**	0.488**	0.507**	0.070	0.633

*n* = 400; **p < 0.01; *p < 0.05 (two-tailed).

##### 3.2.3.2 Confirmatory factor analysis

We conducted a confirmatory factor analysis to examine the discriminant validity of the measured constructs using Amos 27. The results in Table 3 show that the six-factor (error aversion climate, moral disengagement, ego depletion, promotion regulatory focus, prevention regulatory focus, and impoverished leadership) model fits date well (χ^2^/df = 3.08 < 4, RMSEA = 0.072 < 0.08, IFI = 0.869, TLI = 0.856, CFI = 0.869). Comparisons indicate that this model fits better than the other four models, demonstrating good discriminant validity of the measurement tools.

**Table 3:** Model fit results for confirmatory factor analyses.

Model	χ^2^ /df	RMSEA	CFI	IFI	TLI	SRMR	AIC	BIC
Six-factor model(EA; MD; ED; PRF; CRF; LD)	3.08	0.072	0.869	0.869	0.856	0.079	1742.924	2074.215
Five-factor model(CRF + PRF)	3.92	0.086	0.814	0.815	0.798	0.087	2183.808	2495.142
Four-factor model(ED + MD; CRF + PRF)	5.62	0.108	0.703	0.704	0.680	0.105	3078.303	3373.672
Three-factor model(ED + MD + EA; CRF + PRF)	6.54	0.118	0.642	0.643	0.616	0.114	3571.096	3854.490
Tow-factor model (ED + MD + EA + CRF + PRF)	7.24	0.125	0.595	0.597	0.568	0.112	3944.877	4220.288
6. Single-factor model (ED + MD + EA + CRF + PRF + LD)	8.27	0.135	0.527	0.529	0.497	0.122	4495.054	4766.473

n = 400; “EA” indicates error aversion climate; “MD” indicates moral disengagement; “ED” indicates ego depletion; “PRF” indicates prevention regulatory focus; “CRF” indicates “promotion regulatory focus”; “LD” indicates impoverished leadership.

Building on this foundation, we calculated the average variance extracted (AVE) and composite reliability (CR) based on the factor loadings derived from confirmatory factor analysis (CFA) to re-examine the convergent and discriminant validity of the variables. The AVE values all exceeded the recommended threshold of 0.6 (Samy et al., 2021). Similarly, the CR values all surpassed the suggested criterion of 0.6 (Samy et al., 2021). These results reaffirm the convergent validity and discriminant validity of the measurement model used in this study.

To assess potential common method bias, we first conducted Harman’s single-factor test using principal component analysis with unrotated solutions. The results revealed nine factors with eigenvalues greater 1, collectively explaining 68.04% of the total variance. Notably, the first factor accounted for 23.91% of the variance, well below the critical threshold of 40% (Podsakoff et al., 2003), suggesting no severe common method bias in the data.

To further validate this finding, we adopted a more rigorous approach by constructing a bifactor model (Aiken et al., 1991). Specifically, we compared the fit of the original six-factor model with an extended model incorporating an additional method factor (a global factor capturing shared variance across all items). The bifactor model demonstrated acceptable fit indices: χ^2^/df = 2.128, RMSEA = 0.053, IFI = 0.934, TLI = 0.922, CFI = 0.933, and SRMR = 0.041. Compared to the original six-factor CFA model (SRMR = 0.075), the improvements in model fit were marginal: increases in IFI and CFI were less than 0.1, and reductions in RMSEA and SRMR were below 0.05.

##### 3.2.3.3 Hypothesis testing

According to the model structure, data characteristics, and study scenario, we used SPSS 22.0 and the PROCESS macro program to test the hypotheses. All continuous predictor variables were mean-centered prior to constructing interaction terms to reduce multicollinearity and enhance the interpretability of the coefficients (Hayes, 2018).

###### 3.2.3.3.1 Main effect hypothesis testing

This study conducted hierarchical regression analyses to test the hypotheses, as shown in Table 4, the relationship between error aversion climate and impoverished leadership was positive and significant (*β* = 0.405, *p* < 0.001, Model 7). Thus, Hypothesis 1 was supported.

**Table 4:** Regression analysis.

Variables	Moral disengagement			Ego depletion			Impoverished leadership				
	M1	M2	M3	M4	M5	M6	M7	M8	M9	M10	M11
Gender	−0.119**	0.049	0.022	0.007	0.039	−0.022	0.038	0.098	0.02	0.095	0.035
Age	−0.134*	−0.115*	−0.119*	−0.024	−0.032	−0.010	−0.128*	−0.059*	−0.131*	−0.063	−0.12*
Education	0.027	−0.117**	−0.118**	−0.017	0.017	0.008	0.046	0.034	0.061	0.033	0.052
Position	0.071	0.032	0.072	0.019	−0.02	0.014	−0.004	−0.049	−0.027	−0,038	−0.01
EA	0.587***	0.494***	0.582***	0.490***	0.242***	0.478***	0.405***			0.123*	0.246***
PRF		0.183***			0.528***						
CRF			−0.016			0.036					
EA*PRF		0.172***			0.097*						
EA*CRF			0.149***			0.160***					
MD								0.554***		0.480***	
ED									0.442***		0.323***
*F*	36.057	30.681	34.781	17.838	15.732	37.503	13.832	27.397	16.916	24.961	17.838
R2	0.398***	0.415***	0.417***	0.242***	0.266***	0.464***	0.198***	0.329***	0.232***	0.338***	0.242***
ΔR2	0.336***	0.021***	0.028***	0.235***	0.025***	0.009***	0.160***	0.290***	0.194***	0.14***	0.235***

n = 400; “EA” indicates error aversion climate; “MD” indicates moral disengagement; “ED” indicates ego depletion; “PRF” indicates prevention regulatory focus; “CRF” indicates “promotion regulatory focus; “LD” indicates impoverished leadership. ***p < 0.001; **p < 0.01; *p < 0.05 (two-tailed).

###### 3.2.3.3.2 Mediating effect hypothesis testing

This study employed hierarchical regression analysis to test the hypothesis regarding the mediating effect, as shown in Table 4. Error aversion climate is significantly positively related to moral disengagement (*β* = 0.587, *p* < 0.001; Model 1) and ego depletion (*β* = 0.490, *p* < 0.001; Model 4). Moral disengagement (*β* = 0.554, *p* < 0.001; Model 8) and ego depletion (*β* = 0.442, *p* < 0.001; Model 9) are significantly positively related to impoverished leadership. When error aversion climate is included in the equation along with moral disengagement and ego depletion, the effect of error aversion climate on impoverished leadership significantly decreases but remains significant (*β* = 0.123, *p* < 0.05; Model 10), (*β* = 0.246, *p* < 0.001; Model 11). We then adopt the bootstrap method (with sampling for 5,000 times, the same below) to examine the mediating role of moral disengagement and ego depletion. The results are shown in the Table 5. The indirect impact of error aversion climate on impoverished leadership via moral disengagement and ego depletion are significant (effect = 0.334, 95% CI [0.250, 0.422]; effect = 0.188, 95% CI [0.123, 0.259]). Thus, Hypothesis 2 and 3 were supported.

**Table 5:** The bootstrap test on the mediating effect.

Paths and effects	Effects (std_Effect)	SE	95% confidence intervals
Error aversion → Moral disengagement → Impoverished Leadership			
Indirect effect (OLS)	0.334 (0.288)	0.044 (0.038)	[0.250, 0.422]
Indirect effect (HC3)	0.334	0.044	[0.252, 0.419]
Error aversion → Ego depletion → Impoverished leadership			
Indirect effect(OLS)	0.188 (0.162)	0.035 (0.030)	[0.123, 0.259]
Indirect effect(HC3)	0.188	0.034	[0.125, 0.257]

###### 3.2.3.3.3 Moderating effect hypothesis testing

The interaction effect of error aversion climate and prevention regulatory focus(a) (*β* = 0.172, *p* < 0.001, Model 2) and promotion regulatory focus(b) (*β* = 0.149, *p* < 0.001, Model 3) on moral disengagement were positive and significant. The interaction effect of error aversion climate and prevention regulatory focus(a) (*β* = 0.097, *p* < 0.05, Model 5) and promotion regulatory focus(b) (*β* = 0.160, *p* < 0.001, Model 6) on ego depletion were positive and significant.

**Figure 2:**
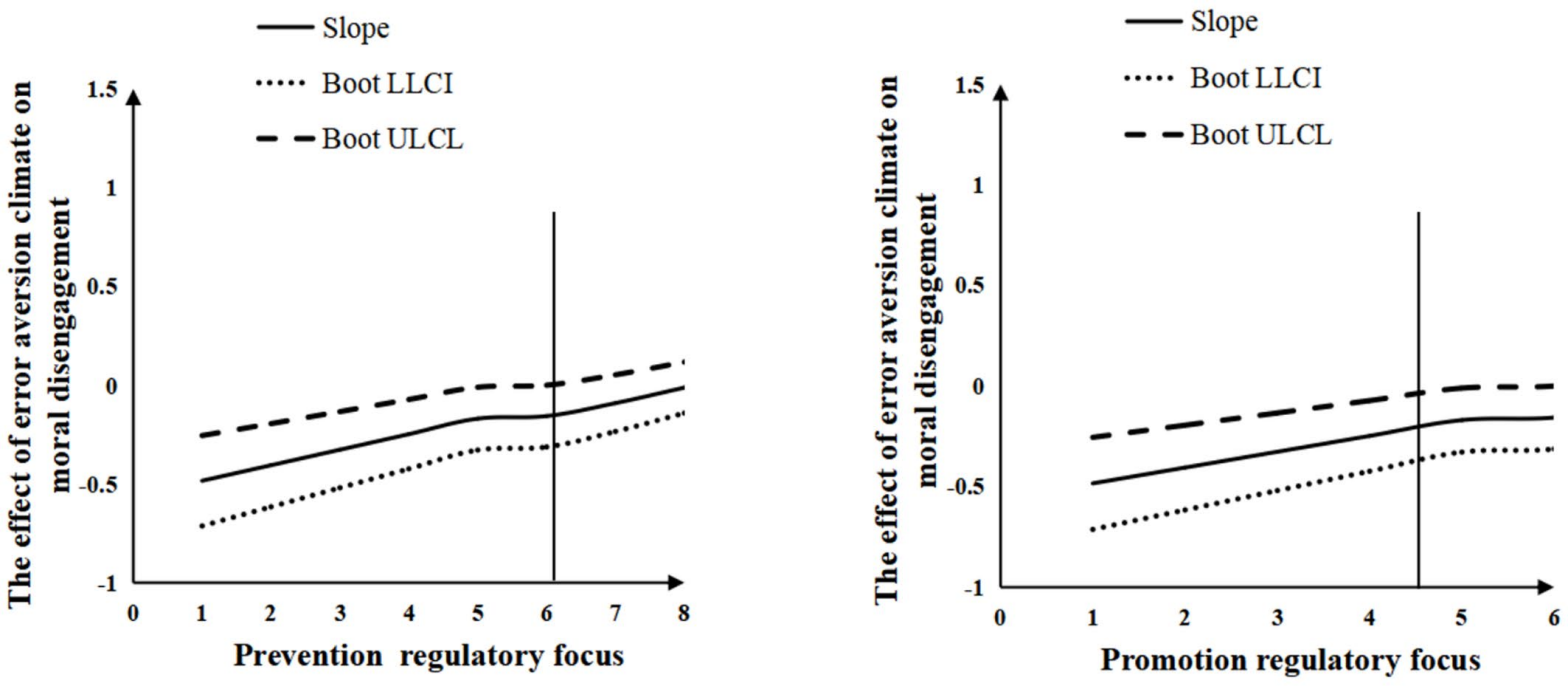
The moderating effect of regulatory focus.

To better illustrate this effect, a JN technique plot was used to visualize the moderating effect of regulatory focus based on its values (Aiken et al., 1991). As shown in Figure 2, with prevention regulatory focus as the horizontal axis and the effect of error aversion climate on moral disengagement as the vertical axis, when prevention regulatory focus scores exceed 6.071, the slope of error aversion climate on moral disengagement becomes significantly greater than zero (*p* < 0.05). Moreover, as prevention regulatory focus scores increase, this slope progressively strengthens. This indicates that error aversion climate exerts a positive predictive effect on moral disengagement when prevention regulatory focus scores surpass 6.071, and this effect intensifies with higher prevention regulatory focus.

Similarly, when promotion regulatory focus is plotted as the horizontal axis, the analysis reveals that when promotion focus scores exceed 4.534, the slope of error aversion climate on moral disengagement becomes significantly positive (*p* < 0.05). As promotion regulatory focus scores rise, the slope further increases, demonstrating that error aversion climate positively predicts moral disengagement under high promotion regulatory focus conditions (scores >4.534), with the effect growing stronger as promotion regulatory focus intensifies. Thus, Hypothesis 4a and 4b were supported.

**Figure 3:**
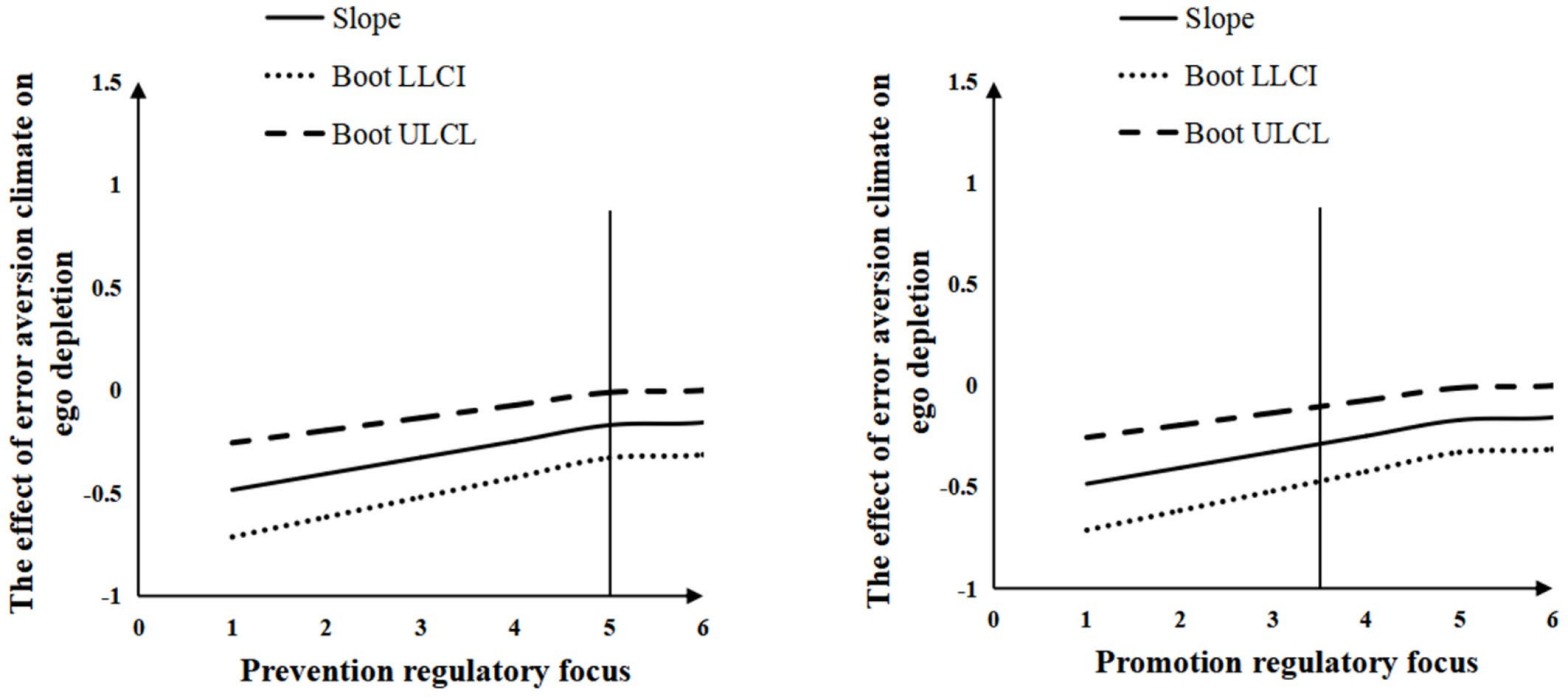
The moderating effect of regulatory focus.

In Figure 3, with prevention regulatory focus as the horizontal axis and the effect of error aversion climate on ego depletion as the vertical axis, when prevention focus scores exceed 4.993, the slope of error aversion climate on ego depletion becomes significantly positive (*p* < 0.05). This slope strengthens progressively with higher prevention regulatory focus scores, indicating that error aversion climate positively predicts ego depletion when prevention regulatory focus scores surpass 4.993, and this predictive effect amplifies as prevention regulatory focus increases.

For the promotion regulatory focus analysis (horizontal axis), when promotion regulatory focus scores exceed 3.413, the slope of error aversion climate on ego depletion becomes significantly greater than zero (*p* < 0.05). As promotion regulatory focus scores rise, the slope continues to increase, suggesting that error aversion climate has a positive predictive effect on ego depletion under high promotion regulatory focus conditions (scores >3.413), with the effect intensifying alongside higher promotion regulatory focus. Thus, Hypothesis 5a and 5b were supported.

###### 3.2.3.3.4 Moderated mediation effect hypothesis testing

This study employs the PROCESS macro for bootstrap analysis to test the moderated mediation effect, with a bootstrap sampling of 5,000 iterations. The results are shown in the Table 6. When prevention regulatory focus(a) and promotion regulatory focus(b) are at a higher level (+1SD), prevention focus path: effects = 0.360, 95% CI = [0.263, 0.463], not including 0; promotion focus path: effects = 0.399, 95% CI = [0.298, 0.512], not including 0. When prevention regulatory focus(a) and promotion regulatory focus(b) are at a lower level (–1SD), prevention regulatory focus path: effects = 0.203, 95% CI = [0.133, 0.283], not including 0; promotion regulatory focus path: effects = 0.264, 95% CI = [0.184, 0.367], not including 0, thus supporting hypotheses H6a and H6b.

**Table 6:** The moderated-mediation effects.

Paths and Effects	Effects	SE	95% confidence intervals
Error aversion climate → Moral disengagement → Impoverished leadership			
Moderated mediation			
Higher prevention regulatory focus (+1SD)	0.360	0.051	[0.263, 0.463]
Lower prevention regulatory focus (−1SD)	0.203	0.038	[0.133, 0.283]
Difference	0.085	0.025	[0.036, 0.136]
Higher promotion regulatory focus (+1SD)	0.399	0.058	[0.298, 0.512]
Lower promotion regulatory focus (−1SD)	0.264	0.046	[0.184, 0.367]
Difference	0.092	0.038	[0.012, 0.163]
Error aversion climate → Ego depletion → Impoverished leadership			
Moderated mediation			
Higher prevention focus (+1SD)	0.116	0.028	[0.072, 0.183]
Lower prevention focus (−1SD)	0.069	0.021	[0.025, 0.109]
Difference	0.032	0.013	[0.009, 0.061]
Higher promotion focus (+1SD)	0.232	0.048	[0.144, 0.330]
Lower promotion focus (−1SD)	0.135	0.031	[0.083, 0.203]
Difference	0.067	0.029	[0.011, 0.124]

Similarly, the differences in the indirect effects of error aversion on impoverished leadership through ego depletion at different levels of prevention regulatory focus and promotion regulatory focus are also significant. Higher level: (prevention regulatory focus path: effects = 0.116, 95% CI = [0.072, 0.183], not including 0); (promotion regulatory focus path: effects = 0.232, 95% CI = [0.144, 0.330], not including 0), Lower level: (prevention regulatory focus path: effects = 0.069, 95% CI = [0.025, 0.109], not including 0); (promotion regulatory focus path: effects = 0.135, 95% CI = [0.083, 0.203], not including 0), thus supporting hypotheses H7a and H7b.

## 4 Discussion and conclusion

### 4.1 Research conclusion

The main conclusions of this study are as follows: Error aversion climate positively affects impoverished leadership; moral disengagement and ego depletion serve as mediators in the relationship between error aversion climate and impoverished leadership respectively; regulatory focus moderates the relationships between error aversion climate and both moral disengagement and ego depletion. Specifically, when leaders have high prevention regulatory focus and high promotion regulatory focus, the positive effect of error aversion climate on moral disengagement is stronger, and the indirect effect of error aversion climate on impoverished leadership is stronger, with moral disengagement mediating the relationship between error aversion climate and impoverished leadership. When leaders have high prevention regulatory focus and high promotion regulatory focus, the positive effect of error aversion climate on ego depletion is stronger, and the indirect effect of error aversion climate on impoverished leadership is stronger, with ego depletion mediating the relationship between error aversion climate and impoverished leadership.

### 4.2 Theoretical implications

The current research possesses three theoretical implications. Firstly, this study employs a combination of scenario surveys and questionnaire experiments to systematically explore the impact of error aversion climate on impoverished leadership, thereby enriching unethical leadership behavior research. Existing leadership studies mostly focus on intense unethical leadership behaviors like abusive supervision and destructive leadership (Bhattacharjee and Sarkar, 2024; Pletzer et al., 2024). However, there is relatively little attention given to conservative leadership behaviors. By focusing on “impoverished leadership,” this research broadens the behavioral spectrum of leadership studies and responds to previous scholars’ calls for more research on leadership avoidance antecedents (Zhang et al., 2022), thereby enriching the field of leadership, particularly in the area of leadership avoidance.

Secondly, this study systematically reveals the dual-path mechanism by which an error aversion climate triggers impoverished leadership by integrating social cognitive theory and conservation of resources theory, making an important expansion to the existing theoretical framework. Firstly, extending from Social cognitive theory, previous research has mostly focused on the explanatory role of moral disengagement in proactive unethical behaviors (such as corruption and deception) (Bandura, 1999). However, this study discovers that moral disengagement also plays a crucial role in passive unethical behaviors (such as impoverished leadership). Specifically, under an error aversion climate, leaders unconsciously rationalize their inaction through cognitive restructuring mechanisms such as “minimizing responsibility” (e.g., “Doing less to avoid mistakes is for the stability of the team”). This process breaks through the traditional social cognitive theory explanatory boundary of “intentional wrongdoing,” revealing the unique role of moral cognitive biases in passive avoidance behaviors. This finding expands the application scenarios of social cognitive theory from “intentional violations” to “unintentional dereliction,” providing a new perspective for understanding the implicit moral risks in organizations.

In the deepening of the resource of conservation theory (COR), this study breaks through the traditional single correlation between resource depletion and job burnout (Hobfoll, 1989), and proposes a new mechanism of “resource conservation inaction.” It is found that the error aversion climate continuously consumes leaders’ psychological resources, forcing them to adopt the strategy of “minimizing input” to preserve the remaining resources, and ultimately leading to impoverished leadership. This mechanism not only extends the theoretical focus of resource of conservation theory from individual mental health to the field of leadership behavior decision-making, but also reveals the central role of resource dynamics in passive behavior choice. Unlike previous studies that emphasized the incentive of resource access to active behavior, this study suggests that resource depletion may indirectly trigger unethical behavior through “defensive withdrawal,” thereby enriching the interpretive dimension of resource of conservation theory in organizational ethics.

Furthermore, the study highlights the synergistic explanatory power of SCT and COR through the construction of a dual-path model. Although other theories, such as moral decision theory, can partially explain the logic of rational choice in which leaders do not act (Zhang et al., 2023), their premise that individuals have full moral consciousness contradicts the reality of “bounded ethicality” (Rees et al., 2019). In contrast, the two-path model in this study covers both unconscious moral cognitive bias (SCT path) and resource-constrained behavioral withdrawal (COR path), which is more suitable for leaders’ decision-making in high-pressure situations.

Finally, this study introduces regulatory focus as a moderating variable to explore the boundary conditions that trigger impoverished leadership. Most existing studies regard prevention regulatory focus and promotion regulatory focus as opposite dimensions (Wang and Wang, 2021), but this study found that the two may produce synergistic effects under specific situations: In error aversion atmosphere, the promotion regulatory focus leaders will accelerate ego-depletion due to excessive pursuit of “zero error” goal, and prevention regulatory focus leaders will intensify moral disengagement due to “error aversion priority” tendency. This finding challenges the simple “promotion-positive/prevention-negative” dichotomy, suggesting that both types of regulatory may work together to reinforce irrational behavior patterns in stressful situations.

### 4.3 Practical implications

This study provides several practical implications for organizational managers to consider.

Firstly, the establishment of a “tolerance-learning” mechanism serves as the cornerstone for balancing error aversion with organizational effectiveness. Central to this approach is the need for organizations to clearly differentiate between “exploratory failures”—reasonable missteps arising from innovation-driven experimentation—and “negligent inaction”—failures stemming from passive avoidance of responsibility. Exploratory failures should be systematically exempted from punitive accountability frameworks, while negligent behaviors must remain subject to strict consequences. To operationalize this distinction, organizations can institutionalize non-punitive error debriefing protocols, such as structured post-failure review sessions, to codify lessons learned from setbacks into actionable knowledge assets. These insights can then be disseminated across teams via internal platforms, transforming failures into shared organizational resources. Concurrently, incentive realignment is critical: leaders who proactively pursue innovation within predefined risk thresholds should be recognized through performance-based rewards (e.g., bonuses, promotions) to reinforce a culture of “intelligent risk-taking.” To prevent misuse, transparent risk-assessment criteria and accountability safeguards must be embedded to ensure error tolerance does not incentivize recklessness. By integrating these strategies, organizations can shift from a fear-driven paralysis rooted in error aversion to a learning-oriented agility that empowers leaders to act decisively while maintaining accountability, thereby breaking the cycle of impoverished leadership.

Secondly, Human resources departments must employ targeted intervention tools to prevent impoverished leadership. By embedding ethical sensitivity metrics (e.g., “propensity for responsibility avoidance,” “frequency of moral disengagement”) into leadership evaluations and integrating 360-degree feedback mechanisms, organizations can achieve early identification of potential risks associated with impoverished leadership. For leaders exhibiting high levels of resource depletion, HR should design personalized resource recovery plans, such as introducing mindfulness-based stress reduction programs or offering temporary role detachment periods, to help them rebuild psychological resources and restore decision-making capacity. Additionally, scenario-based simulation assessments should be incorporated into leadership selection processes. These simulations could replicate high error aversion climate (e.g., rapid decision-making during crises) to observe candidates’ behavioral patterns under pressure, prioritizing those who demonstrate the ability to balance risk-taking with accountability. This multi-pronged approach ensures proactive mitigation of impoverished leadership while fostering resilience and ethical accountability in organizational leaders.

Finally, leadership development strategies grounded in regulatory focus theory can maximize organizational adaptability. For promotion regulatory focus leaders, training should emphasize resource conservation tactics, such as breaking high-risk tasks into manageable sub-goals to reduce cognitive load per decision, while equipping them with resilience-building techniques (e.g., cognitive reappraisal) to sustain decision-making capacity under high-pressure scenarios. For prevention regulatory focus leaders, interventions must target cognitive bias correction—for instance, systematically exposing them to case studies highlighting team failures caused by inaction to amplify accountability awareness. A phased approach can be implemented, starting with low-stakes decisions and incrementally increasing responsibility weight (e.g., from approving routine budgets to crisis response planning), thereby dismantling the “inaction-as-safety” mindset.

### 4.4 Limitation and future direction

Although this study makes some contributions to the existing research, it has several limitations, which provide suggestions and opportunities for future research.

Firstly, While the experimental design of Study 1 effectively controlled variables through scenario simulations, its reliance on a sample primarily drawn from university student populations introduces potential limitations to external validity. Students may systematically differ from actual corporate managers in terms of workplace experience, risk perception, and organizational accountability. For instance, students’ lack of practical managerial experience could lead to under-sensitivity to accountability risks, potentially skewing experimental effects (e.g., overestimating or underestimating relationships in real-world corporate settings). To enhance generalizability, future research should replicate the experimental protocol with mid-level corporate managers as participants and conduct comparative analyses to assess cross-sample consistency.

In the part of this study 2 that investigates the impact of an error aversion climate on leadership avoidance through a questionnaire survey, only cross-sectional data were collected. However, due to the limitations of cross-sectional data in explaining dynamic relationships between variables, this study cannot test the dynamic impact of an error aversion climate on impoverished leadership. Therefore, future research could be designed as longitudinal studies, such as using experience sampling methods or collecting data at multiple time points to explore the role of time in the process by which an error aversion climate affects impoverished leadership. Additionally, to collect authentic data without causing inconvenience to the participants, this study used projective methods to measure leadership avoidance. Future research could measure leadership avoidance through other evaluations, such as by having subordinates or superiors of the leaders provide assessments through paired methods.

Secondly, While this study constructs its theoretical model based on social cognitive theory and conservation of resources theory, it acknowledges the potential validity of alternative explanatory frameworks. For instance, social exchange theory could further elucidate leaders’ risk–reward trade-off mechanisms (Ahmad et al., 2023). In error aversion climate, leaders may perceive an effort-reward imbalance (e.g., anticipated penalties for proactive accountability outweigh potential rewards), prompting psychological withdrawal as a self-protective strategy. Future research could develop integrated models to compare the explanatory power of distinct theoretical pathways, while employing cross-level analyses to examine how organizational systems (e.g., error tolerant climate, institutional support) interact with individual perceptions to shape leadership behaviors.

Additionally, The study did not test measurement invariance across demographic or organizational subgroups (e.g., senior vs. grassroots managers). While our sample demonstrated homogeneity in key variables (e.g., education, tenure), future research should establish configural, metric, and scalar invariance to ensure cross-group comparability. The sample of this study is from Chinese organizations, and cultural values (such as collectivism, high uncertainty avoidance) may influence the results. In the future, the theoretical model should be validated in a multicultural context to enhance its universality.

Finally, while this study identified the moderating role of regulatory focus in the relationship between an error aversion climate and impoverished leadership, it did not consider the moderating effects of other personality traits, such as the Dark Triad and the Big Five personality traits. The Dark Triad consists of psychopathy, narcissism, and Machiavellianism. Leaders with Machiavellian or psychopathic traits often disregard others’ feelings and traditional morality for their own benefit, while narcissistic leaders tend to be self-centered and are commonly found in leadership positions (Schlegel and Mortillaro, 2019). Conscientiousness, a dimension of the Big Five personality traits, is associated with high achievement motivation in leaders (Umphress et al., 2020). Given this, the Dark Triad and Big Five personality traits may also have moderating effects. Therefore, future research could explore these personality traits to examine the boundary conditions in the relationship between an error aversion climate and impoverished leadership.

## Data Availability

The original contributions presented in the study are included in the article/Supplementary material, further inquiries can be directed to the corresponding author/s.
